# Melt Spinnability Comparison of Mechanically and Chemically Recycled Polyamide 6 for Plastic Waste Reuse

**DOI:** 10.3390/polym16223152

**Published:** 2024-11-12

**Authors:** Kyuhyun Kim, Minsoo Kim, Yerim Kim, Jinhyeong Kim, Jihwan Lim, Woojin Lee, Han Seong Kim, Dong-Hyun Cho, Jaejun Lee, Sejin Choi

**Affiliations:** 1 School of Chemical Engineering, Pusan National University, Busan 46241, Republic of Korea; 2 Department of Organic Material Science and Engineering, Pusan National University, Busan 46241, Republic of Korea; 3 Institute of Advanced Organic Materials, Pusan National University, Busan 46241, Republic of Korea; 4 Department of Aerospace Engineering, Pusan National University, Busan 46241, Republic of Korea; 5 Department of Polymer Science and Engineering, Pusan National University, Busan 46241, Republic of Korea

**Keywords:** recycled polymer, recycled fiber, nylon fiber, molecular orientation, XRD, crystallinity, phase difference, hot press, FT-IR analysis

## Abstract

With the increasing volume of synthetic fiber waste, interest in plastic reuse technologies has grown. To address this issue, physical and chemical recycling techniques for polyamide, a major component of textile waste, have been developed. This study investigates the remelting and reforming properties of four types of pristine and recycled polyamide 6, focusing on how the microstructural arrangement of recycled polyamides affects polymer fiber formation. DSC and FT-IR were used to determine the thermal properties and chemical composition of the reformed thin films. Differences in the elongation behavior of molten fibers during the spinning process were also observed, and the morphology of the resulting fibers was examined via SEM. Birefringence analysis revealed that the uniformity of the molecular structure greatly influenced differences in the re-fiberization process, suggesting that chemically recycled polyamide is the most suitable material for re-fiberization with its high structural similarity to pristine polyamide.

## 1. Introduction

The rapid spread of fast fashion has led to a significant increase in textile waste and environmental pollution [1,2,3]. The 2.1 billion tons of annual greenhouse gases emitted during the life cycle of synthetic fiber products contribute to climate change, while soil contamination and microplastic generation from landfill and marine disposal present severe environmental challenges [4]. Approximately 70% of the synthetic fiber waste generated each year consists of polyamide and polyester, amounting to around 25 million tons [5,6,7]. As such, the development of recycling and reuse technologies for synthetic fiber waste across various industries is a critical issue for sustainable development.

The recycling technologies for polymer waste, such as fibers, can be categorized into mechanical recycling and chemical recycling [8,9,10]. Mechanical recycling involves grinding polymer waste into pellets or flakes, which is followed by remelting and reforming processes to produce new products. While this method is simple and cost effective, it has drawbacks such as difficulty in removing contaminants and a decline in material quality due to molecular degradation from thermal and mechanical stress [11,12]. Chemical recycling, on the other hand, refers to the depolymerization process, where the waste is chemically broken down into monomers or co-monomers, which are then re-polymerized. This method allows for the recovery of monomers with very high purity, making it possible to obtain high-quality materials with properties almost identical to those of pristine raw materials [13].

Nylon, a synthetic polymer first developed in the 1930s, is a thermoplastic polyamide widely used in various industrial fields due to its beneficial physical properties, such as high strength and elasticity [14,15,16]. In particular, products requiring high mechanical properties, such as ropes, fishing nets, and gear, extensively utilize single-material nylon 6 fibers because of their excellent durability and chemical resistance [17,18,19]. Unlike many mixed-material plastic products that are difficult to separate, nylon-based fishing nets and gear could be more suitable for recycling. However, long-term exposure to harsh environments leads to degradation and contamination, making conventional recycling processes challenging. As a result, research on chemical recycling methods for nylon reuse has been actively pursued [20,21,22].

Polyamide fibers can be produced through various methods, with melt processing being the most commonly utilized [23]. Melt spinning involves melting thermoplastic polymers and extruding them through a spinneret at temperatures above their melting point, after which the fiber shape is fully formed [24,25]. High-strength, highly oriented fibers are produced through a relatively simple drawing process, and an environmental benefit is that no organic solvents are required [26,27]. Nonetheless, to successfully manufacture fibers through the continuous deformation processes of extruding, drawing, and winding, several challenges must be overcome, one of which is thermal decomposition and fiber breakage that inevitably occur during melt spinning. For polyester and polyamide, which make up a large portion of melt-spun products, material properties such as molecular weight, polydispersity index, and melt flow index, as well as process parameters such as temperature, pressure, and speed, have been optimized to withstand the harsh conditions of high temperatures and high tension [28,29,30]. However, research on the re-fiberization of recycled materials, which are particularly vulnerable to thermal decomposition, has been very limited, and even this research has been mainly focused on polyester [31,32,33,34].

Therefore, the formability and spinnability of recycled polyamides are essential characteristics that must be demonstrated to support the developments of future applications. In this study, we investigated the formability of polyamide using four different types of pristine and recycled polyamide 6 (PA6), and we examined the effects of the microstructure of recycled PA6 on spinnability and fiber formation. To determine thermal characteristics and chemical composition, while excluding the influence of the microscopic molecular alignment, reformed thin films produced via hot pressing were used. Subsequently, differences in elongational flow during melt spinning were observed for each material. The correlation between re-fiberization behavior and the uniformity of molecular entanglement was explained through phase difference analysis. To the best of our knowledge, only a few studies have compared the properties of pristine and recycled PA6 and their melt spinnability. We expect that this report on the melt spinning of recycled polyamide will provide valuable data for addressing the urgent issue of plastic waste recycling and expanding potential applications.

## 2. Materials and Methods

### 2.1. Materials

**Figure 1:**
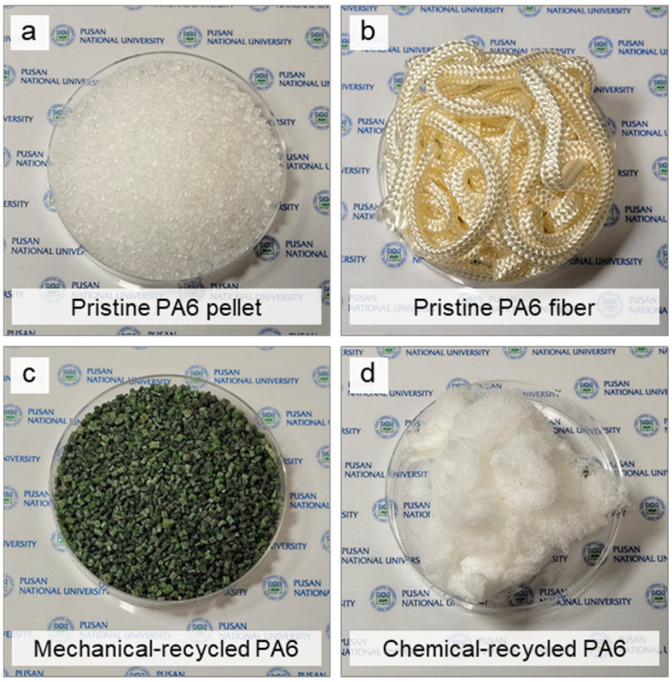
Various PA6 materials used for the forming process: (**a**) pristine PA6 pellets, (**b**) pristine PA6 fibers, (**c**) mechanical-recycled PA6, and (**d**) chemical-recycled PA6.

To investigate the re-fiberization of recycled PA6, one pristine (unrecycled) material and three recycled materials were prepared. The pristine PA6 (pellets, density 1.084 g mL^−1^, T_m_ 228.5 °C, T_g_ 62.5 °C) was purchased from Sigma-Aldrich Inc. (St. Louis, MO, USA) (Figure 1a). Fishing net fiber made from pristine PA6 (Figure 1b), mechanically recycled PA6 from used fishing nets (Figure 1c), and chemically recycled PA6 (Figure 1d) were provided by KTI Co. (Busan, Republic of Korea), as materials for reforming. These four materials were named in the following order: p-PA, p-PF, mr-PA, and cr-PA, respectively. All materials were used without additional chemical modification.

### 2.2. Preparation of Thin Films

**Figure 2:**
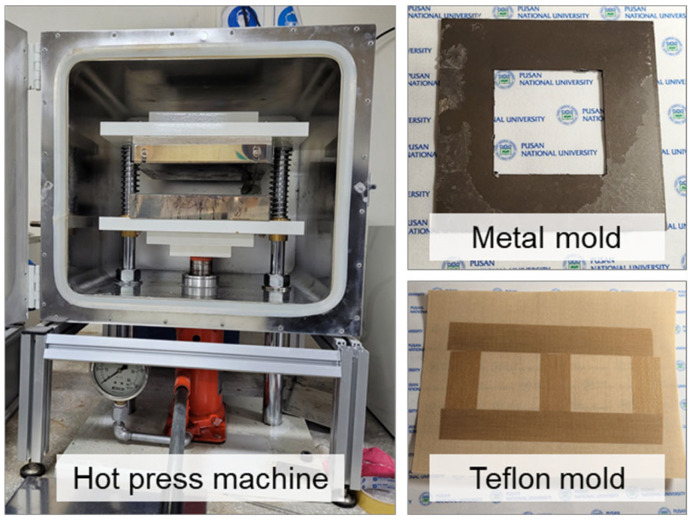
Hot press machine and forming mold made from different materials.

Unlike p-PA, p-PF, mr-PA, and cr-PA have undergone more than one thermal process, resulting in significantly different molecular orientations and crystalline structures, making simple comparisons of physical properties, thermal characteristics, and formability across the materials inappropriate. Therefore, a hot press (Ocean Science Co., Uiwang, Republic of Korea) was used to prepare the specimens for analysis and to assess thermal formability, as shown in Figure 2. A custom-made mold was used to control the thickness. To prepare the test specimens, 1.5 g of each pristine and recycled material was compressed at a temperature of 260 °C and a pressure of 2.5 MPa. The p-PA, p-PF, and mr-PA were pre-heated for 630, 470, and 510 s, respectively, and then compressed for 30 s, while the cr-PA was pre-heated for 150 s before being compressed for 60 s.

### 2.3. Setup of Melt Spinning Process

**Figure 3:**
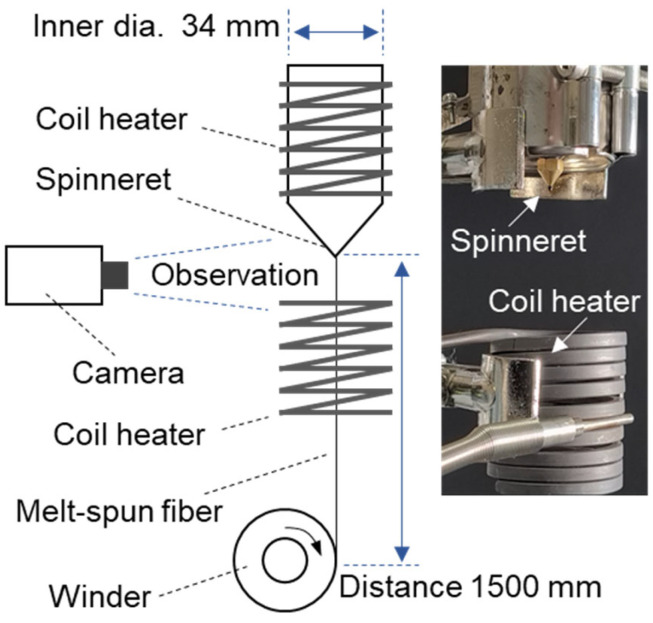
Schematic of the melt spinning setup.

The melt spinning of pristine and recycled PA6 was performed using a custom-made metal cylinder and a winder (Ocean Science Co., Uiwang, Republic of Korea). The metal cylinder with an inner diameter of 34 mm was coupled with a coil heater to melt the polymer materials, as shown in Figure 3. Below the spinneret, with an inner diameter of 0.3 mm, another coil heater was placed to maintain a high-temperature environment for the extruded melt. The cylinder temperature was maintained at 230 °C, and the lower coil heater was set to 200 °C. The polymer melt was extruded under a constant air pressure of 0.03 MPa using an air compressor (EWS30, G&P, Seoul, Republic of Korea). The winder, positioned 1500 mm away from the spinneret, was used to wind the fibers at various rotational speeds ranging from 300 to 2000 rpm. The melt spinning conditions, including the linear speed information of the winder, are shown in Table 1.

### 2.4. Characterization

The thermal properties of the four types of PA6 materials were measured using a differential scanning calorimeter (DSC8500, Perkin Elmer Co., Waltham, MA, USA) under a nitrogen atmosphere. The samples were heated and cooled in a temperature range of 0 to 250 °C with a heating rate of 5 °C min^−1^. The chemical composition of the recycled materials was analyzed using Fourier-transform infrared spectroscopy (FT-IR, IRAffinity-1, Shimadzu Korea Co., Seoul, Republic of Korea) in the wavenumber range of 800 to 3700 cm^−1^. The morphology of the produced fibers was observed using a polarized microscope (Eclipse LV100 POL, Nikon Co., Tokyo, Japan) and a scanning electron microscope (SNE-4500M, SEC Co., Ltd., Suwon, Republic of Korea), and the phase difference was confirmed using an image analysis system. The crystalline structure of each PA6 fiber was compared through X-ray diffraction (XRD, Xpert 3, Malvern Panalytical Ins., Malvern, UK).

## 3. Results and Discussion

**Figure 4:**
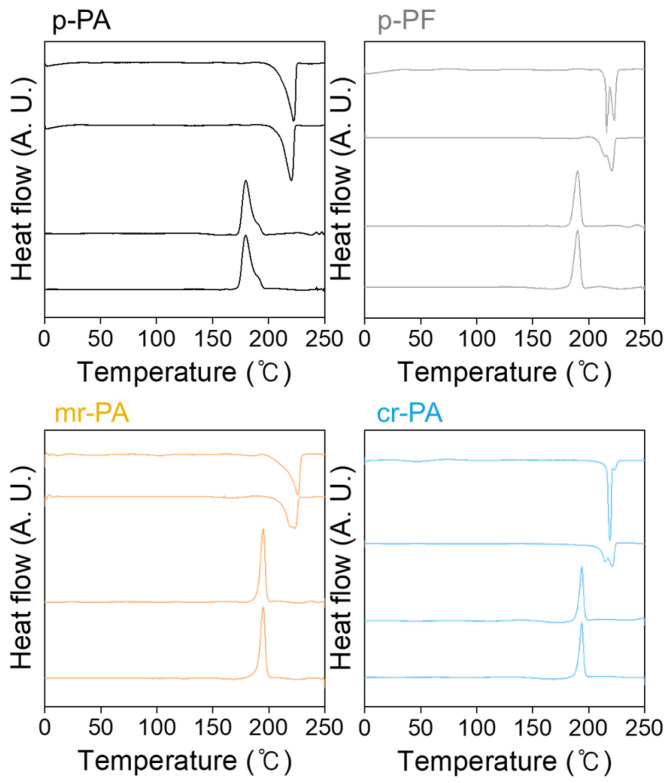
Thermal properties of pristine and recycled PA6 materials.

For the successful melt processing of PA6, it is essential to determine the appropriate processing temperatures. The melting conditions required to produce thermally compressed specimens from p-PA, p-PF, mr-PA, and cr-PA were investigated. The endothermic peaks, shown in the DSC thermogram in Figure 4, represent the melting behavior of each PA6 material. Based on the onset temperature at which melting begins, the melting temperatures (T_m_) of p-PA and mr-PA are identified as 215 °C and 217 °C, respectively. However, the fiber-type materials exhibit multiple peaks due to the mixed structure of semi-crystalline regions and highly oriented molecules [35,36]. For p-PF, endothermic peaks are observed at 215 °C and 220 °C, and for cr-PA, peaks are identified at 217 °C and 222 °C, indicating slightly higher melting points compared to pellet-type materials. The broad endothermic peaks commonly seen during the second heating process suggest the melting of crystals generated during the DSC measurement. Consequently, the melting points of both pristine and recycled PA6 were found to be within the range of approximately 215 to 225 °C.

**Figure 5:**
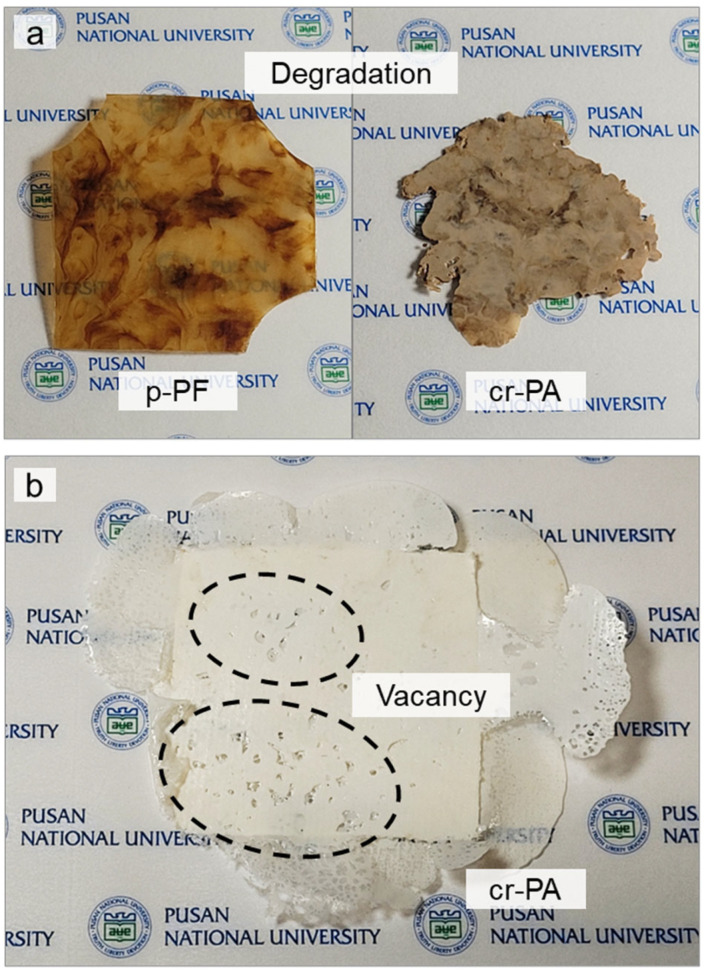
(**a**) Thermal decomposition and (**b**) vacancy defects caused during hot pressing.

**Figure 6:**
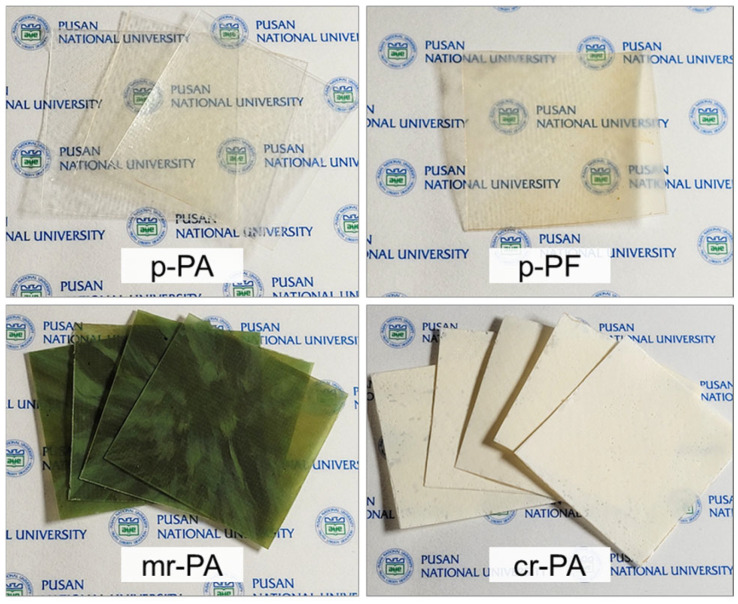
Hot-pressed films of pristine and recycled PA6 materials.

To accurately compare the inherent material properties of various polyamides, it is necessary to eliminate the molecular structural differences between the pellet-type samples with spherulites and the fiber samples with highly oriented molecules. Thus, thin films were prepared through thermal compression using a hot press machine. One of the issues encountered during the preparation of hot melt film was thermal decomposition occurring before the material melted, which was particularly frequent in the fiber-type materials, such as p-PF and cr-PA (Figure 5a). Stretching of the polymer induces a molecular packing structure, enhancing secondary bonding, which generally strengthens the material properties. In particular, fibers achieve highly aligned and packed molecular structures through extreme elongation. The highly developed molecular orientation of the fiber samples seemed to increase intermolecular bonding strength, causing the intramolecular covalent bonds to break before the secondary bonds were fully released by thermal energy [37,38]. Furthermore, despite having the same mass as the pellet-type samples, the fiber samples contained more pores, requiring the control of bubbles or vacancies that occur during the hot-pressing process, as shown in Figure 5b. To address these issues, a pre-heating process was applied to ensure that each PA6 material was sufficiently melted and stabilized before compression, as shown in Table 1. As a result, thin films with thicknesses of 350, 410, 340, and 440 μm for p-PA, p-PF, mr-PA, and cr-PA, respectively, were successfully produced, as shown in Figure 6.

**Figure 7:**
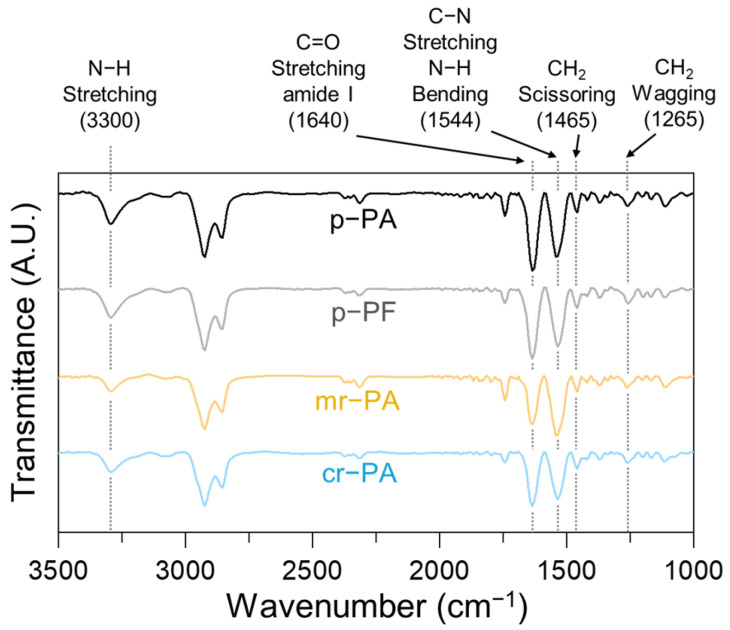
FT-IR spectra of pristine and recycled PA6 materials.

Since the provided PA6 materials have different prior histories, it is necessary to determine the chemical composition and the presence of impurities for a reasonable comparison of their formability. Figure 7 shows the FT-IR spectra of p-PA, p-PF, mr-PA, and cr-PA. In all materials, characteristic peaks of the polyamide group are observed, including the N-H stretching at 3300 cm^−1^, C=O amide stretching at 1640 cm^−1^, and C-N stretching and N-H bending at 1544 cm^−1^. The characteristic peaks of PA6 at 1265 cm^−1^ and 1465 cm^−1^, corresponding to CH₂ wagging and CH₂ scissoring, respectively, are also confirmed [39]. Furthermore, no peaks associated with impurities are present, indicating that all specimens consist of pure PA6.

The fiber formation process, which can be explained by the development of molecular alignment and crystallization during the viscous flow of molten polymers, is determined by the continuity and evenness of the produced fibers. To enable the continuous discharge of the melt from the spinneret, the previously extruded molten polymer needs to drag the next polymer molecule by means of intermolecular interactions, specifically the molecular entanglement of the polymer melts [40,41]. The drag force among the entangled molecules must be able to withstand the high winding tension, meaning differences in the microstructural state of the polymer lead to variations in spinnability, even with the same material. Therefore, we observed the spinning behavior at winding speeds ranging from 300 to 2000 rpm to evaluate the potential for re-fiberization of PA6 with different histories. A cylinder with an inner diameter of 34 mm, designed to minimize the temperature gradient from the inner wall to the center, was used to ensure a homogeneous melt. The cylinder temperature was consistently set to 230 °C for all materials based on the thermal property results.

**Figure 8:**
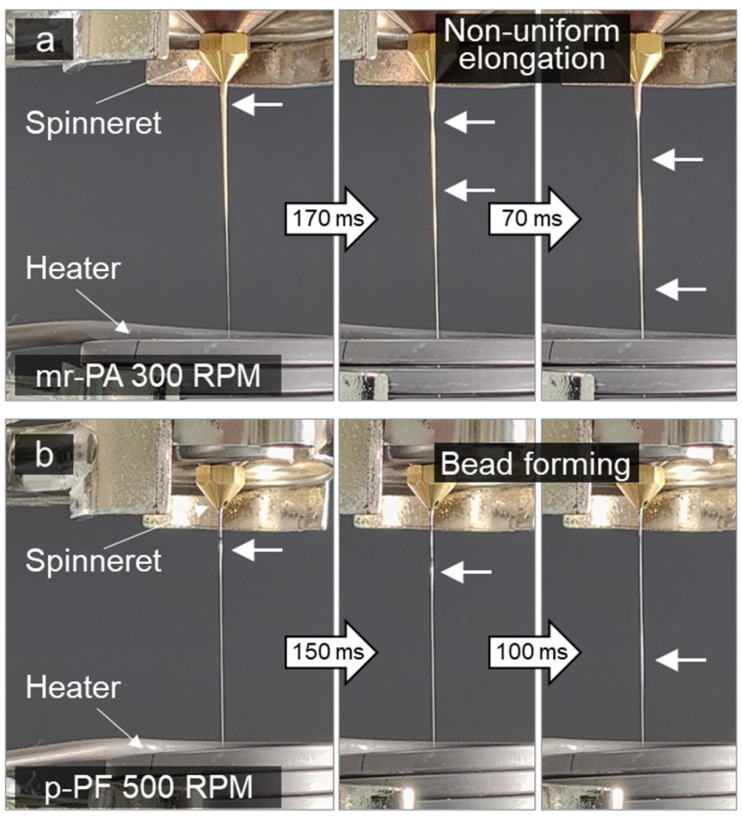
Sequential images of unstable melt spinning, showing the occurrence of (**a**) non-uniform elongation in mr-PA fiber, and (**b**) bead formation in p-PF fiber.

Figure 8 displays sequential images of the process in which the extruded molten material is elongated by winding tension when mr-PA and p-PF are melt-spun at 300 and 500 rpm, respectively. In Figure 8a, it can be observed that the molten fiber of mr-PA, moving toward the winder, experiences uneven elongation flow in certain areas rather than extending uniformly across its length. Likewise, the p-PF shows more frequent occurrences of uneven elongation, resulting in bead formation, as shown in Figure 8b. It is expected that mr-PA, which has been mechanically recycled, has undergone more remelting processes, leading to a diverse polymer chain distribution. Moreover, although the fiber-type p-PF is made from pristine PA6, simultaneous melting and thermal decomposition occur during the remelting process, similar to what occurs during mechanical recycling, due to the stacking structure of the highly oriented molecules, as mentioned above. The wide distribution of polymer chains hinders the uniformity of molecular entanglement, which plays an important role in withstanding winding tension, causing uneven stress transmission within the molten fiber. Fluctuations in molecular drag speed lead to the formation of uneven fibers or beads instead of continuous fibers. Particularly, when the winding speed increases, the fiber fails to overcome the high tension and eventually breaks.

**Figure 9:**
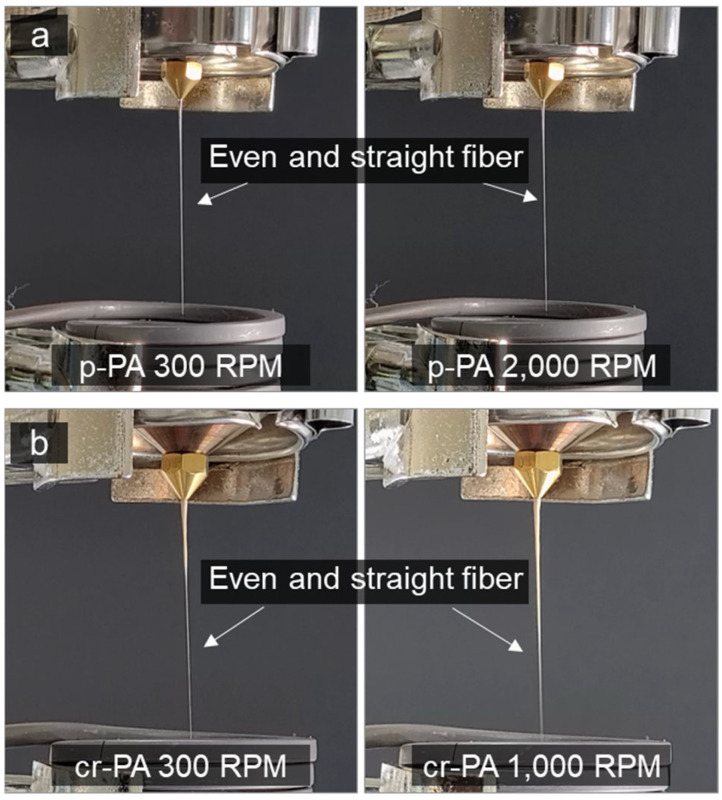
Stable melt spinning of (**a**) p-PA and (**b**) cr-PA at the minimum and maximum winding speeds.

While fibers were formed for mr-PA and p-PF only at the lower winding speeds of 300 and 500 rpm, respectively, stable melt spinning was achieved with p-PA and cr-PA at the maximum winding speeds of 2000 and 1000 rpm, respectively. Figure 9a,b present images of p-PA and cr-PA fibers being spun at the minimum and maximum winding speeds. Both p-PA and cr-PA formed even and straight molten fibers, which is believed to result from the uniform molecular distribution that enables the formation of uniform molecular entanglement within the microstructure. The uniform molecular structure of the polymer allows for even stress transmission under high tension, facilitating the smooth dispersion and dissipation of internal stress. Despite the difference in winding speeds for p-PA and cr-PA, both were able to form even fibers at high winding speeds without breakage or bead formation. The spinnability of each material at various winding speeds is summarized in Table 2. Note that for p-PF, due to its very low melt viscosity, spinning could not be performed at 300 rpm because the extrusion rate exceeded the winding speed; however, it was possible at 500 rpm.

**Figure 10:**
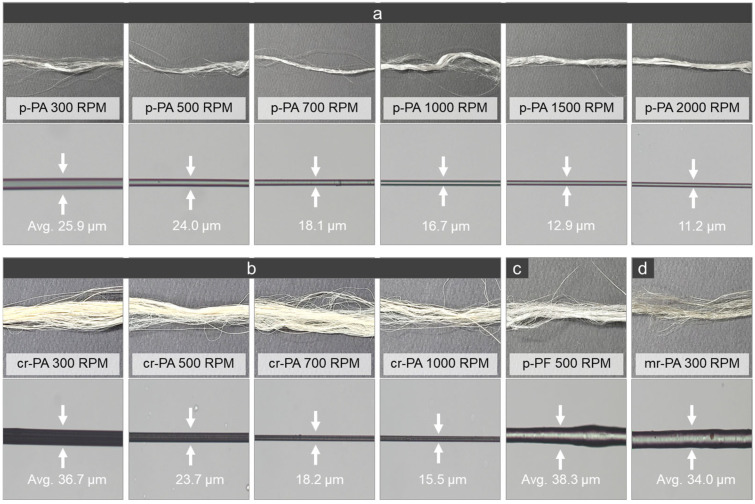
Actual and optical microscope images of melt spun fibers: (**a**) p-PA, (**b**) cr-PA, (**c**) p-PF, and (**d**) mr-PA.

Figure 10 shows the actual and optical microscope images of p-PA, p-PF, mr-PA, and cr-PA fibers produced at various winding speeds. As the speed increases from 300 to 2000 rpm, the diameter of the p-PA fiber continuously decreases from 25.9 to 11.2 μm, as seen in Figure 10a. With the increase in winding speed, the tensile force also increases, resulting in fibers with a thinner diameter and more even surface. In particular, molecular alignment is expected to improve at higher winding speeds, enhancing the structural stability of the fiber. The cr-PA fibers show a similar tendency with the diameter decreasing from 36.7 to 15.5 μm at 300 to 1000 rpm (Figure 10b). The similar diameters of p-PA and cr-PA at the same speeds suggest that cr-PA has a similar development of chain orientation to p-PA. In contrast, p-PF and mr-PA fibers were only spun at lower speeds due to their inability to withstand the winding tension, resulting from the uneven distribution of their molecular structures. The insufficiently elongated p-PF and mr-PA fibers had relatively high average diameters of 38.3 μm and 34.0 μm, respectively, and exhibited uneven surfaces (Figure 10c,d).

**Figure 11:**
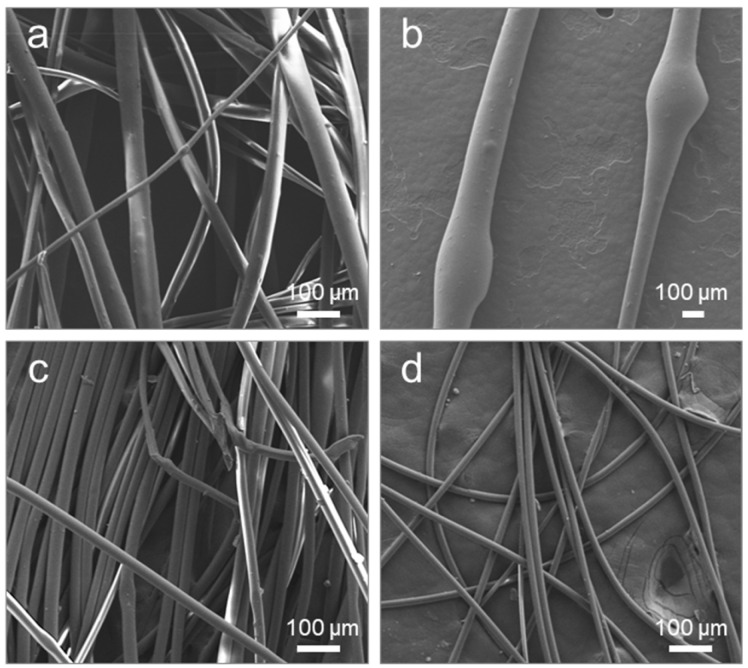
SEM images of melt-spun fibers: (**a**) mr-PA at 300 rpm, (**b**) p-PF at 500 rpm, (**c**) p-PA at 2000 rpm, and (**d**) cr-PA at 1000 rpm. Scale bar: 100 µm.

The non-uniform elongation during the melt spinning process of p-PF and mr-PA resulted in a wide fiber diameter distribution, as shown in Figure 11a, with bead formation clearly visible in Figure 11b. In contrast, the p-PA and cr-PA fibers fabricated through stable and high-speed spinning displayed relatively thin diameters, more uniform distributions, and smoother surfaces (Figure 11c,d). Despite being made of the same PA6, there was a noticeable difference in elongation behavior between p-PF/mr-PA and p-PA/cr-PA, leading to macroscopic shape changes in the resulting fibers. As previously mentioned, the different morphologies are believed to come from the molecular arrangement, specifically the uniformity of the entanglement. To demonstrate that a regular molecular distribution is a key factor in re-fiberization, phase difference analysis was conducted.

The phase difference obtained from the birefringence of transmitted light is known as an indicator of the material’s microstructure [42,43]. A uniform and consistent phase difference across the measurement surface indicates that the path of the transmitted light remains uniform regardless of the part of the object [44]. Thus, it can represent the homogeneity of the microstructure that affects the path of the transmitted light [45,46].

**Figure 12:**
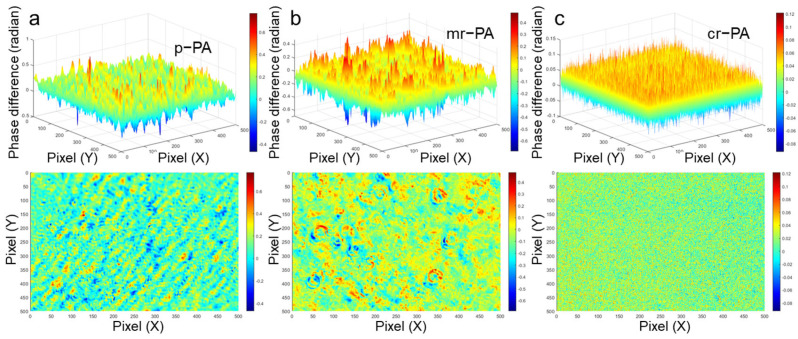
Visualization of phase difference distribution based on birefringence values measured along the cross-sections of (**a**) p-PA, (**b**) mr-PA, and (**c**) cr-PA.

Figure 12 presents a visualized image of the phase difference values measured along specific cross-sections of the p-PA, mr-PA, and cr-PA materials, which are mapped onto the XY plane by corresponding the phase difference values to color for clarity. In Figure 12a,c, the uniform color distribution of p-PA and cr-PA indicates consistent phase differences in the transmitted light, suggesting a homogeneous molecular arrangement in both samples. In contrast, Figure 12b shows a larger variation in phase difference values and a more irregular distribution of phase differences, suggesting that the macromolecules in mr-PA have microstructures that are more unevenly distributed. Such non-uniform microstructures can lead to discontinuous stress transmission when spinning tension is applied, potentially resulting in partial elongation or fiber breakage. Therefore, a highly uniform molecular entanglement is essential for stable fiber spinning, and it appears that pristine and chemically recycled materials have microstructures more favorable for spinnability compared to mechanically recycled materials.

**Figure 13:**
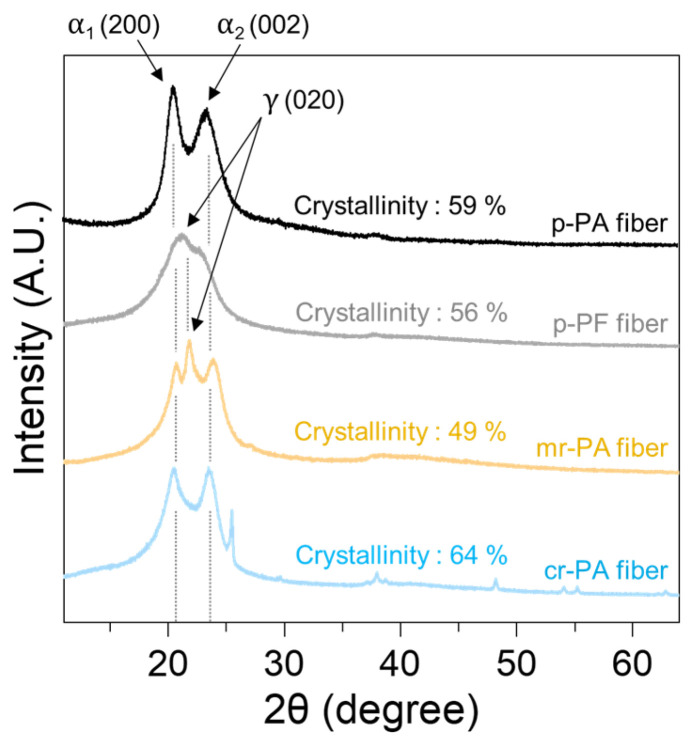
XRD spectrum of PA6 fibers produced with various pristine and recycled materials.

Figure 13 shows the XRD spectra representing the crystallinity of cr-PA, mr-PA, p-PF, and p-PA fibers produced through the melt spinning process. PA6 is a typical semi-crystalline polymer with the main peaks corresponding to the (200), (002), (020) planes being α₁, α₂, γ, respectively [15,47]. The α-phase is frequently observed in polyamide crystals, which is characterized by a stable molecular arrangement based on regular hydrogen bonding between molecules [48]. On the other hand, the γ-phase is related to a looser molecular arrangement and asymmetric hydrogen bonding, caused by structural deformation and increased amorphous regions, resulting in lower mechanical strength and thermal stability [49,50]. In p-PA and cr-PA, which possess uniform molecular entanglement, rapid disentanglement and subsequent molecular alignment occurred at high winding speeds, leading to the formation of stable crystalline structures [51,52,53,54]. Strong α₁ and α₂ peaks indicate the development of the α-crystalline phase in PA6. Note that the peak observed around 25 degrees in the XRD pattern of cr-PA corresponds to titanium dioxide nanoparticles [55], which are commonly used as a delustering agent in fibers. However, the α₁ and α₂ peaks in mr-PA and p-PF fibers were relatively weak with the γ peak being dominant. The non-uniform elongation of mr-PA and p-PF during the spinning process disrupted the development of well-aligned polymer molecules. Consequently, the insufficient viscous flow resistance of the molten fibers hindered the formation of highly crystalline fibers. These results suggest that the microstructure of cr-PA is more advantageous for regenerating fibers with spinnability, morphological characteristics, and physical properties similar to pristine PA6 compared to p-PF and mr-PA.

## 4. Conclusions

This study focused on the melt spinning of fibers using p-PA, p-PF, mr-PA, and cr-PA materials. DSC analysis confirmed that the melting points of the four types of PA 6 were within the range of approximately 230 °C, reflecting differences in the molecular structures and processing histories of each material. After successfully preparing the thin film specimens based on their thermal properties, FT-IR analysis demonstrated that all samples were composed of pure PA 6 free from impurities. Subsequently, the elongation behavior of molten fibers during the spinning process was observed for all materials. While mr-PA and p-PF exhibited unstable spinnability at lower winding speeds due to the uneven distribution of molecular structures, p-PA and cr-PA displayed stable spinnability even at higher winding speeds thanks to their uniform molecular distribution. Optical microscopy and SEM images of the produced fibers revealed that mr-PA and p-PF formed beads, had irregular surfaces, and showed wide diameter distributions. In contrast, p-PA and cr-PA fibers displayed smooth surfaces and uniform diameter distributions. Phase difference analysis further identified that the uniform molecular microstructure in p-PA and cr-PA contributed to their superior spinnability. Finally, XRD analysis indicated that cr-PA exhibited a well-developed crystalline structure similar to that of pristine p-PA, suggesting that chemically recycled PA6 is a favorable material for re-fiberization.

## Figures and Tables

**Table 1:** Melt spinning conditions for various PA6 materials.

Discharge pressure (MPa)	0.03					
Cylinder temperature (°C)	230					
Coil heater (°C)	200					
Winding speed (rpm)	300	500	700	1000	1500	2000
Linear velocity (m min^−1^)	170	283	396	565	848	1130

**Table 2:** Spinnability at various winding speeds for pristine and recycled PA6 materials.

Winding Speed (rpm)	300	500	700	1000	1500	2000
p-PA	Spinnable	Spinnable	Spinnable	Spinnable	Spinnable	Spinnable
p-PF	Not feasible	Spinnable	-	-	-	-
mr-PA	Spinnable	-	-	-	-	-
cr-PA	Spinnable	Spinnable	Spinnable	Spinnable	-	-

## Data Availability

The data presented in this study are available on request from the corresponding author.
